# Glucagon-Like Peptide-1 Receptor Agonists in Neurodegenerative Diseases: A Comprehensive Review

**DOI:** 10.7759/cureus.92441

**Published:** 2025-09-16

**Authors:** Ruba Ibrahim, Aya Kambal, Mohammed A Abdelmajeed

**Affiliations:** 1 Medicine and Surgery, University of Medical Sciences and Technology (UMST), Khartoum, SDN

**Keywords:** alzheimer's disease, glucagon-like peptide-1 receptor agonists (glp-1 ras), neurodegenerative diseases, neuroprotection, parkinson's disease

## Abstract

There is a steadily increasing global burden of neurodegenerative diseases (NDs) such as dementia, including Alzheimer’s disease (AD), and movement disorders like Parkinson’s disease (PD), which emphasizes the importance of disease-modifying therapy (as opposed to symptomatic therapy). This review considers a new disease-modifying strategy through the so-called glucagon-like peptide-1 receptor agonists (GLP-1 RAs) in these NDs, and the mechanistic rationale and evidence base. The review considers a multi-targeting strategy, translational issues, and a future research agenda. Translational challenges for these agents are also discussed, particularly the need to understand how the timing of treatment may influence outcomes. The review also considers unwanted side effects like unintended weight loss, which may compromise “at-risk” patient groups. In conclusion, GLP-1 RAs are an intriguing multitarget treatment option for people with NDs, and future research should focus on optimizing treatment for clinical trials, evolved agonists that can penetrate the central nervous system (CNS), and combination therapies.

## Introduction and background

Neurodegenerative diseases (NDs) represent a growing global concern that impacts quality of life and places a large burden on healthcare systems [1]. There are more than 55 million people with dementia worldwide, and the majority of these individuals have AD, with estimates set to rise to 150 million by 2050 [2]. In the US alone, 7.2 million people aged 65 and older have AD, with estimates increasing to 13.8 million by 2060 [3]. PD affects more than 10 million people worldwide, making it the second most common ND, and prevalence is expected to double by 2050 [4].

The pathophysiology of NDs is complex and involves interrelated cellular and molecular events. In AD, neurocognitive function is disrupted by intracellular aggregates of hyperphosphorylated tau (neurofibrillary tangles), and extracellular amyloid-beta (Aβ) plaques [5]. In PD, patients experience progressive loss of dopaminergic neurons in the substantia nigra with consequent reductions in dopamine and motor symptoms such as tremors, rigidity, bradykinesia, and postural instability, and also non-motor symptoms including cognitive decline and mood disturbances [6].

Currently in AD, cholinesterase inhibitors and NMDA receptor antagonists offer brief cognitive symptomatic improvement [7], while newer therapies targeting Aβ, including lecanemab and donanemab, report modest disease-modifying effects, but long-term outcomes and safety remain unknown [8]. In PD, levodopa and dopamine agonists may improve motor function but similarly have long-term side effects, including dyskinesias and loss of efficacy over time [1]. To date, none of these approaches is truly neuroprotective or neurorestorative, which represents a pressing unmet need in the field.

The use of glucagon-like peptide-1 (GLP-1) receptor agonists (RAs) as potential therapies for NDs has been receiving attention lately. GLP-1 is an incretin hormone that modulates glucose homeostasis and helps promote energy balance by stimulating insulin, inhibiting glucagon, slowing gastric emptying, and regulating appetite [9]. GLP-1 RAs have consistent use as therapies for type 2 diabetes (T2DM) and obesity, and these agents also convey cardiovascular and renal benefits [10].

Metabolic dysfunction (T2DM, hyperinsulinemia, obesity) is a risk factor for neurodegeneration, especially in AD and PD, reflecting shared pathways including oxidative stress, chronic inflammation, mitochondrial dysfunction, and impaired insulin signaling - hence sometimes termed “Type 3 diabetes” in AD [9]. GLP-1 receptors have high expression in the brain, including the neocortex and hippocampus, which suggests GLP-1 RAs may modulate neuronal activity directly and push beyond effects on metabolic function only [11].

In this review, we explore both preclinical and clinical evidence for the use of GLP-1 RAs in NDs, highlight the mechanistic rationale and therapeutic opportunity, discuss challenges to translation, and end with speculations on the future.

## Review

GLP 1 receptor distribution in the brain

The central nervous system (CNS) represents a critical and increasingly studied target for the implications of GLP-1 and its analogues. While the primary source of endogenous GLP-1 remains the L endocrine cells of the gastrointestinal tract [12], a significant and functionally distinct pool of GLP-1 is synthesized by preproglucagon (PPG) neurons, predominantly located within the caudal brainstem - namely within the nucleus of tractus solitarius [13]. The circulating gut-derived GLP-1 can subsequently enter the bloodstream and readily cross the blood-brain barrier (BBB) to exert its effect centrally [14]. Similarly, preclinical studies indicate that select GLP-1 RAs, including liraglutide, semaglutide, and exenatide, demonstrate varying degrees of CNS penetrance, enabling direct central action [15,16]. In contrast to the relatively localized central site of synthesis, GLP-1 receptors (GLP-1Rs), conversely, are extensively distributed in the brain [13,17].

*Specific Brain Regions and Cellular Localization* 

GLP‑1 receptors (GLP-1Rs) are densely expressed in numerous key brain regions critical for neurodegenerative pathology and related functions. Their distribution is summarized in Table 1. Beyond specific anatomical regions, ultrastructural studies consistently confirm the predominant neuronal expression of GLP-1Rs on cell bodies, dendrites, and presynaptic terminals in vivo [18]. This precise neuronal localization underscores their direct involvement in modulating synaptic transmission, neuronal excitability, and synaptic plasticity - mechanisms fundamentally important in neurodegenerative processes.

**Table 1:** GLP-1 Receptor Expression in Different Brain Regions. Table credit: Aya Kambal

Brain Region	Key Nuclei/Structures	Primary Functions Linked to GLP-1R Expression	References
Brainstem	Nucleus of the Tractus Solitarius, Area Postrema	Autonomic control, appetite regulation	^[13,14,19]^
Hypothalamus	Arcuate Nucleus, Paraventricular Nucleus, Dorsomedial Hypothalamic Nucleus	Appetite control, energy balance, neuroendocrine regulation	^[13,18]^
Hippocampus	Dentate Gyrus (Mossy Cells, ventral hippocampus emphasis)	Learning, memory, neuronal activity	^[14,20]^
Midbrain	Ventral Tegmental Area, Substantia Nigra (dopaminergic neurons)	Reward pathways, motor control	^[14,21]^
Cortex	Frontal, Prefrontal Cortex	Cognitive functions, executive function	^[17,22]^
Striatum	Caudate, Putamen	Motor control, reward pathways	^[13,14]^

Cross-Species Consistency and Functional Implications

Non-human primate studies consistently report GLP-1R expression in neuroanatomical regions, analogous to those found in rodents and humans [14]. This cross-species conservation suggests that central GLP-1R signaling is fundamentally important for regulating conserved physiological functions, including autonomic control, energy homeostasis, and higher-order cognitive function. The consistent distribution across species further strengthens the translational relevance of preclinical findings and supports the ongoing investigation of GLP-1 RAs as a therapeutic strategy for human neurodegenerative conditions.

Overall, the extensive, region-specific, and cell-type-specific distribution of GLP-1Rs throughout the brain provides a robust anatomical and functional foundation for the diverse neurobiological actions of GLP-1 and its agonists. Elucidating the precise mechanisms by which GLP-1 RAs leverage this intricate receptor distribution to exert their therapeutic effects in neurodegeneration remains a critical area of ongoing research.

GLP-1 agonists and neurodegeneration: A mechanistic overview

Glucagon-like peptide-1 receptor agonists (GLP-1 RAs) activate several intracellular signaling cascades critical for neuronal survival, metabolic regulation, and neuroinflammation control. Through multitarget modulation, they address key pathological processes in neurodegeneration. The following overview examines some of the major pathways.

Signaling Pathways

cAMP-PKA-cAMP response element-binding (CREB) Pathway: The GLP-1 receptor (GLP-1R), a Gs protein-coupled receptor, mediates its effects by elevating intracellular cyclic adenosine monophosphate (cAMP) levels, which subsequently activate protein kinase A (PKA) [13,23,24]. PKA then phosphorylates cAMP response element-binding (CREB) protein, a pivotal transcription factor that increases the expression of neurotrophic and anti-apoptotic genes, such as brain-derived neurotrophic factor (BDNF) and B-cell lymphoma 2 (Bcl-2) [13,24,25]. Activation of this axis enhances neuronal survival, promotes synaptic plasticity, and improves the resilience of neurons to excitotoxic stress, as observed in various in vitro models, including hippocampal HT22 cells [13,23-25].

PI3K-Akt-GSK‑3β/mTOR Signaling: GLP‑1R activation triggers the phosphoinositide 3-kinase (PI3K) pathway, leading to the downstream phosphorylation of Akt (also known as protein kinase B). Activated Akt inhibits pro-apoptotic factors such as Bad and glycogen synthase kinase 3 beta (GSK‑3β), which is implicated in tau hyperphosphorylation, a hallmark of Alzheimer’s disease (AD). Concurrently, Akt stimulates the mammalian target of rapamycin (mTOR) pathway, supporting protein synthesis, cell growth, and overall metabolic health [2,25]. This comprehensive pathway preserves neuronal viability, supports mitochondrial integrity, and attenuates various degenerative processes [2,26,27].

MAPK/ERK Pathway: Through direct GLP‑1R activation and potentially via Akt-dependent crosstalk, GLP‑1 RAs stimulate the mitogen-activated protein kinase/extracellular signal-regulated kinase (MAPK/ERK) signaling pathway (Ras-Raf-MEK-ERK cascade) [26,28]. This pathway promotes the transcription of genes involved in neuronal growth, repair, and differentiation [28]. The activation of MAPK/ERK signaling consequently enhances neuroplasticity and facilitates regeneration, effects observed in various in vivo models of early brain injury [25,26].

Metabolic Regulation

AMPK Activation and Energy Balance: GLP‑1R signaling leads to the activation of AMP-activated protein kinase (AMPK) via CaMKK2. Activated AMPK stimulates PGC-1α dependent mitochondrial biogenesis, which is crucial for maintaining cellular energy production and function [29]. Additionally, AMPK activation reduces the activity of beta-site amyloid precursor protein cleaving enzyme 1 (BACE1), an enzyme involved in the production of amyloid-beta (Aβ) peptides [29]. It also enhances the phagocytic capacity of microglia, promoting the clearance of pathological protein aggregates. These comprehensive metabolic effects contribute to an improved cellular energy balance, reduced oxidative stress, and enhanced clearance of neurotoxic protein aggregates within the brain [29,30].

*Mitochondrial Health and Autophagy Modulation* 

In addition to the discussed mechanisms, GLP-1 RAs provide neuroprotective benefits, through their impact on mitochondrial function, promoting cellular autophagy. Activation of AMPK, as previously mentioned contributes to mitochondrial biogenesis (the process of generating new mitochondria) [29], and primarily also improves mitochondrial dynamics, achieving a healthy and flexible mitochondrial network with the means to meet neuronal energy demands [30]. The more favorable mitochondrial health - more ATP produced and importantly, lowering of oxidative stress due to the dysfunction of mitochondria and one of the prominent causes of neuronal damage - directly counteracts the mitochondrial dysfunction, that is relatively common in neurodegenerative constructs [30,31]. More importantly, GLP-1R activation can induce autophagy through pathways including PI3K-Akt and AMPK. Dictated by the functions that cellular autophagy represents the cellular degradation and recycling of damaged organelles, aggregated proteins, and misfolded proteins [24,26]. Together, with GLP-1 RAs selective and effective elimination of aggregated proteins and dysfunctional mitochondria GLP-1 RAs support maintenance of cellular homeostasis and contribute to limiting over accumulation of toxic material that are hallmarks present in neurodegenerative disorders [23,30,32]. 

GLP-1 R as modulators of neuroinflammation

Growing evidence suggests that GLP-1 receptor agonists (GLP-1 RAs) have significant modulatory effects on neuroinflammation by acting on important cellular and molecular components in the CNS. They have been able to induce an anti-inflammatory response through the changes in microglial actions, astrocytes, and the neurovascular unit, all of which play a role in maintaining homeostasis of the CNS in normal health. Unlike the physiological distribution of GLP-1Rs in neurons, expression of these receptors in the glial cells of the CNS is increasingly found to be inducible in the setting of injury or disease rather than constitutionally expressed [33].

Most notably, GLP-1 RAs work on microglia to induce a phenotypic shift from an M1-like pro-inflammatory phenotype to an M2-like anti-inflammatory phenotype, suppress NF-κB activation, and inhibit inflammasome signaling, thereby reducing the release of cytokines such as TNF-α and IL-1β while enhancing anti-inflammatory mediators like IL-10 [34-36]. There is also blocking of NLRP3 inflammasome, thereby diminishing the production of reactive oxygen species (ROS) and mitigating downstream neurotoxicity [9].

In astrocytes, GLP1 RAs prevent their differentiation into a reactive A1 neurotoxic state and alter astrocytic inflammasomes to limit the overall neuroinflammatory cascade [37]. They also help restore astrocytic functions critical for blood-brain barrier (BBB) homeostasis, through AQP4 polarization that is crucial for aiding the clearance of neurotoxic metabolites and supporting the preservation of neurovascular integrity [38].

Finally, through stabilization of I-kappa-B-alpha (IκBα), there is downregulation of inflammasome-mediated cytokine release and the overall reduction of the pro-inflammatory cytokine environment in the CNS, further protecting the BBB [39]. Detailed cellular mechanisms are summarized in Table 2.

**Table 2:** Summary of Cellular and Molecular Mechanisms of GLP-1 Receptor Agonists in Modulating Neuroinflammation. Table credit: Aya Kambal

Cell/Target	Mechanism of Action	Functional Outcome	Relevant Sources
Microglia	Induce shift from M1 (pro-inflammatory) → M2 (anti-inflammatory) phenotype; inhibit NF-κB activation; block NLRP3 inflammasome; ↓ TNF-α, IL-1β; ↑ IL-10	Reduced pro-inflammatory cytokines, ↓ ROS, enhanced tissue repair	[9, 34–36]
Astrocytes	Prevent transition to A1 neurotoxic phenotype; modulate NLRP2 inflammasome; restore aquaporin-4 (AQP4) polarization	Prevent transition to A1 neurotoxic phenotype; modulate NLRP2 inflammasome; restore aquaporin-4 (AQP4) polarization	[37, 38]
Blood–Brain Barrier (BBB)	Stabilize IκBα to inhibit NF-κB nuclear translocation; downregulate MMPs and adhesion molecules.	Preserved BBB integrity; reduced immune cell infiltration into CNS	[39]
Overall Effect	Coordinate suppression of neuroinflammation and restoration of CNS homeostasis	Neuroprotection and potential therapeutic benefit in neurodegenerative diseases	[9, 34–39]

**Figure 1:**
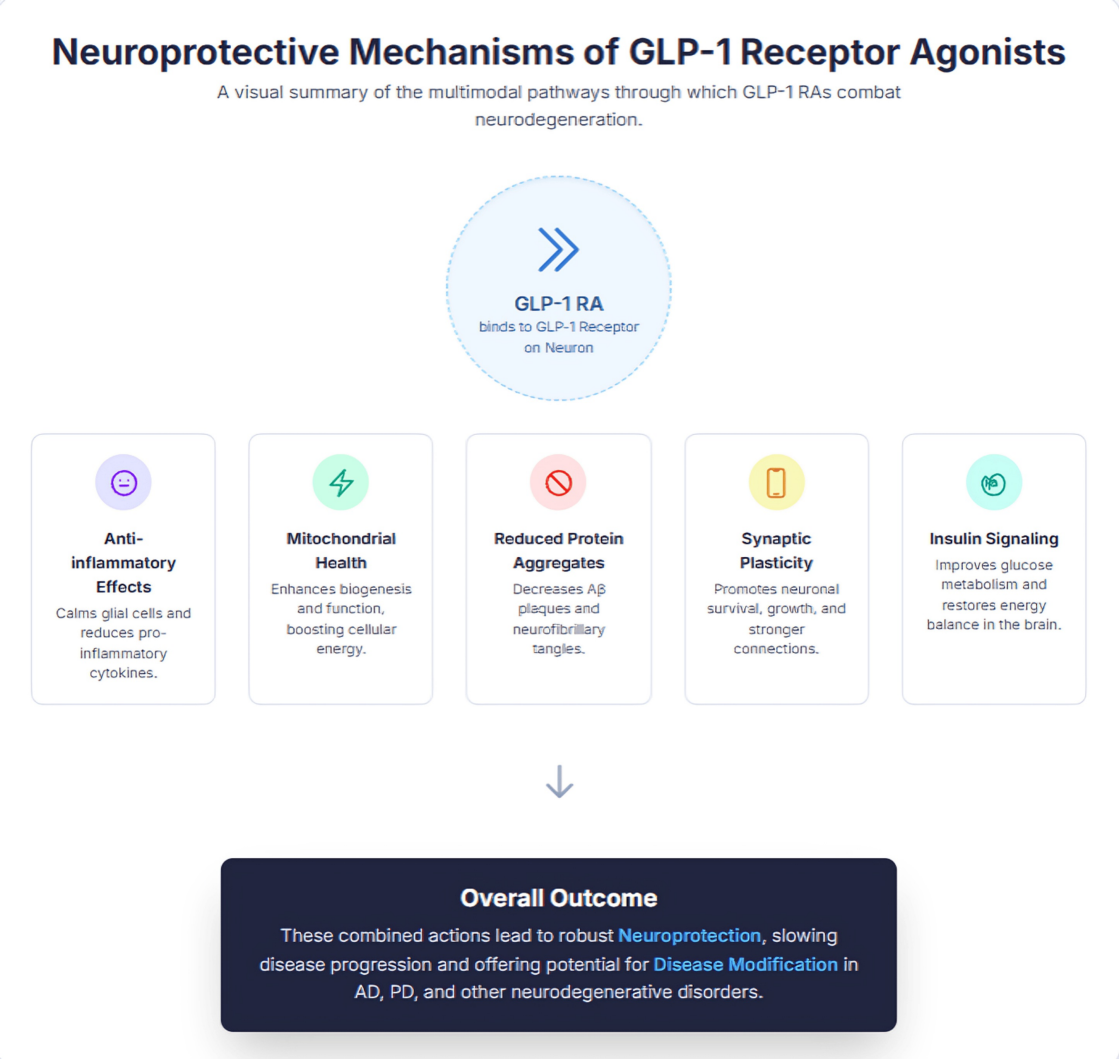
A Visual Summary of the Multimodal Pathways Through which GLP-1 Receptor Agonists Combat Neurodegeneration. Figure credit: Ruba Ibrahim using Canvas.

The evidence supports that GLP-1 RAs contribute to neuroprotection through the modulation of the activation states of microglia toward M2 and injury repair, dampening the reactivity of astrocytes toward neuroinflammatory insult, blocking important signaling pathways pertaining to inflammation, and maintaining a functional BBB. These collective actions will all contribute to dampened neuroinflammatory responses, and the re-establishment of CNS homeostasis and GLP-1 RAs could be translated into the clinical treatment of neuroinflammation. To add on, the figure below is a visual summary of the multimodal pathways that have been mentioned above (Figure 1).

Crosstalk between insulin resistance and neurodegeneration

Insulin signaling is crucial for energy use, mitogenesis, neuronal growth, synapse formation, inhibition of apoptosis, and more. Insulin attaches itself to the receptor’s α-subunit. Tyrosine kinase phosphorylation of the β-subunit is triggered by this. Several second messenger pathways can then be triggered as a result. The insulin receptor being activated leads to gene expression of proteins necessary for cell development, synapse formation, and cell maintenance and repair, which is activated by the Shc-MAP kinase pathway. Neurotransmission is directly impacted by insulin receptor activation, which also prepares synapses for the generation of long-term neural transmission potentiation. Changes in neurotransmission will impact cognitive functions, information processing, and memory formation [40].

According to preclinical studies, Animal models of AD and PD have altered insulin signaling and a variety of downstream effects that contribute to the disease. One significant discovery in recent years has been that people with AD and PD also have desensitized insulin signaling in their brains. The insulin and insulin-like growth factor 1 (IGF-1) receptor, insulin receptor substrates 1 and 2 (IRS1/2), and important second messenger kinases like Akt and mTOR were all found to be inactivated in patient brain tissue, which is comparable to what is seen peripherally in diabetes. AD has even been called “type 3 diabetes” [40].

Low IQ, dementia, neurodegeneration, brain aging, brain shrinkage, and cognitive decline are all brought on by hyperglycemia. It seems that brain injury from hyperglycemia is a multifactorial phenomenon involving a multitude of pathophysiologic mechanisms such as vascular pathology, oxidative stress, neuroinflammation, mitochondrial dysfunction, apoptosis, deficits in neurotrophic factors, AChE activation, altered neurotransmitters, deficits in regeneration of brain tissue, malfunction of the brain glymphatic system, tau hyperphosphorylation, accumulation of amyloid β, and neurodegeneration [41].

Insulin resistance (IR) promotes neuroinflammation in the brain. Chronically high blood sugar levels and an unhealthy blood lipid imbalance are implications of IR that might harm and enhance the permeability of the blood-brain barrier. Microglia are triggered into a pro-inflammatory state in response to BBB disruption, free fatty acids, and elevated blood glucose. This can result in the release of cytokines, downstream neuroinflammatory cascades, and reactive astrogliosis. The BBB is then further damaged by neuroinflammation, which increases its permeability. This increased permeability raises the brain’s already high blood glucose levels and feeds a vicious cycle of neuroinflammation brought on by hyperglycemia. Vascular malfunction (which has been connected to dementia that coexists with ND), increased NO generation, oxidative stress, and the resulting neuroinflammation are further neuroinflammatory effects of brain IR [9].

Furthermore, by promoting excessive mitochondrial respiration in the brain, which raises ROS production and subsequently activates NF-κB, cytokine production, and related neuroinflammatory signaling cascades, chronic hyperglycemia and IR exacerbate neuroinflammation [42].

GLP-1 agonists in Alzheimer’s disease

Numerous studies have shown that people with diabetes who take GLP-1RA have a decreased incidence of all-cause dementia than those who do not. More research is required to determine if this decrease is the result of improved cardiovascular and diabetes risk factors or AD-specific neuroprotection [31].

In mice, ligarglutide restored the IR loss and cognitive impairment brought on by AβOs (amyloid beta oligomers). The findings also suggested that ligarglutide may have some neuroprotective effects in NHPs. While liraglutide reversed cognitive damage and loss of IR mRNA content and completely protected mice’s brains from AβO-induced cognitive impairment, we also observed that the treatment provided partial protection against receptor and synapse loss in some brain regions examined in NHPs [43].

A study comparing placebo to exenatide found that with the probable exception of the exenatide group’s increase in attention and short-term memory, neuropsychological tests were essentially the same between the exenatide and placebo groups. It’s important to note that both groups did exhibit notable declines over time in the MMSE, CDR-sob, temporal lobe volume by MRI volumetrics, and other AD-related measures, indicating that cognitive decline did occur over the study period. Therefore, even though they were sensitive to cognitive deterioration, there are no trends in the total cognitive scores, clinical evaluations, or traditional biomarkers that support the idea that exenatide is affecting the course of early clinical AD. MRI imaging revealed no patterns or variations in the two groups’ atrophy progression that might be related to the neuroprotective effects of exenatide [44].

GLP-1 agonists in Parkinson’s disease

According to a large population-based cohort study, the incidence of Parkinson’s disease (PD) in individuals with type 2 diabetes varies significantly depending on the diabetes medication they receive. Users of GLP-1 RAs and DPP4 inhibitors had a 36-60% reduced incidence of PD than users of other oral antidiabetic medications. Age, smoking status, and the length of time a person had diabetes before the index date were all taken into account when calculating the estimated association. When compared to other antidiabetic drug exposures, the results of additional analyses that censored follow-up time at the moment of stopping the index and comparator drugs demonstrated a clear protective connection between current exposure to GTZ, DPP4, and GLP-1 and PD [45].

The impact of lixisenatide versus placebo on the development of motor impairment in PD patients was evaluated in a double-blind, randomized, placebo-controlled study. At 12 months, the lixisenatide group’s MDS-UPDRS part III scores (which range from 0 to 132, with higher scores indicating greater motor disability) had changed by −0.04 points, indicating improvement, while the placebo group’s scores had changed by + 3.04 points, indicating worsening disability. Accordingly, lixisenatide at 12 months reduced the progression of motor impairment compared to a placebo in this phase 2 trial, but was linked to serious gastrointestinal adverse effects [46].

Another study demonstrated neuroprotective effects of GLP-1 analogues in MPTP-induced PD mice. Drugs were administered once daily for 14 days, and MPTP was administered once daily for 7 days. Liraglutide (25 nmol/kg), lixisenatide (10 nmol/kg), and exendin-4 (10 nmol/kg) were compared; exendin-4 did not exhibit any protective effects at the selected dose. In the substantia nigra and basal ganglia, liraglutide and lixisenatide both demonstrated effects in preventing MPTP-induced motor impairment (as measured by the Rotarod, open-field locomotion, and catalepsy test); lowering tyrosine hydroxylase (TH) levels (dopamine synthesis); decreasing the pro-apoptotic signaling molecule BAX; and raising the anti-apoptotic signaling molecule B-cell lymphoma-2. The findings indicate that liraglutide and lixisenatide both outperform exendin-4 in this investigation and have potential as new PD treatments [47].

GLP-1 agonists in other neurodegenerative diseases (Amyotrophic lateral sclerosis (ALS), Huntington’s disease (HD))

There are currently no clinical trials looking into the use of GRA in Huntington’s disease (HD). Preclinical research has now shown that GRA can ameliorate abnormal characteristics in HD animals. Liraglutide therapy has been demonstrated to decrease ROS, increase cell survival, and improve insulin signaling in neurons overexpressing mutant huntingtin (HTT) proteins [48]. Furthermore, by activating the AMPK pathway, liraglutide stimulates the autophagy process in neural cells, increasing the cells’ ability to remove mutant HTT protein clumps [47]. Liraglutide therapy reduced 3-nitropropionic acid (3-NP)-induced striatal structural and histological damage and enhanced rat cognitive function in a 3-nitropropionic acid (3-NP) HD model. In 3-NP-treated rats, protective effects are ascribed to changes in several pathways, such as the BDNF/TrkB pathway, insulin signaling, Nrf2 and anti-apoptotic pathways, and neuroinflammation pathways [49].

Liraglutide treatment did not, however, improve motor function in SOD1G93A and TAR DNA-binding protein [43] (TDP-43)Q331K transgenic Amyotrophic lateral sclerosis (ALS) mice models, according to earlier investigations [50]. In the spinal cord of ALS mouse models, liraglutide did not reduce motor neuron loss, glial activation, or other characteristic pathological alterations, nor did it increase longevity or postpone the onset of terminal disease phases [49]. Additionally, a case report reported that an ALS patient’s condition progressed more quickly after receiving [51].

Comparative effectiveness with GLP-1 agonists and other neuroprotective agents

GLP-1 RAs have a different profile of action than any available neurotherapeutics. In the case of PD, current treatments - dopamine agonists and MAO-B inhibitors - mainly treat the symptoms of PD and have limited disease-modifying effects [1]. While initial trials of exenatide suggested the possibility of disease modification with longer-term improvements in motor function [52], the larger, later phase 3 EXENATIDE-PD3 trial showed no sizable improvement for the exenatide cohort [53]. However, we can note that other GLP-1 RAs, like lixisenatide, may have some ability to slow the progression of motor symptoms, with liraglutide showing improvement in non-motor symptoms, quality of life, and functional benefits [6]. Further, the mechanisms of action are novel relative to the symptomatic nature of PD drugs, which makes it feasible for GLP-1 RAs to modify the underlying processes of the disease.

In the case of AD, current pharmacological interventions (cholinesterase inhibitors and NMDA receptor antagonists) are limited to symptom management. In contrast, preclinical models of GLP-1 analogues have focused on the pathologies underlying the dual processes of amyloid-beta (Aβ) accumulation and tau hyperphosphorylation, which current symptomatic agents do not directly address. While there is limited availability of agents that show evidence of preventative treatment or disease modification, emerging studies mention certain anti-amyloid monoclonal antibodies as a potential mainstay of treatment in early AD. Lecanemab is one such drug that gained FDA approval for treatment in July 2023. However, the serious adverse events of these agents, such as amyloid-related imaging abnormalities, that could potentially lead to life-threatening outcomes, are a major concern [54].

In addition to targeting specific diseases, GLP-1 RAs are known to have broad neuroprotective actions that incorporate strong antioxidative actions through the reduction of ROS and mitochondrial support. This could give GLP-1 RAs an advantage over others, such as coenzyme Q10 or creatine, which, while promising in preclinical trials, have largely failed in clinical trials for PD [55].

The multifactorial nature of NDs indicates that combination therapy may be beneficial. By their broad actions, GLP-1 RAs seem to also be promoted as synergistic agents with other drugs. For example, in preclinical models of PD, neuroprotective and anti-inflammatory effects were increased when exenatide was combined with multiple agents, including sodium phenylbutyrate and tauroursodeoxycholic acid [55]. Thus, there is merit in expanding combinatorial strategies that take advantage of co-administering GLP-1 RAs with anti-inflammatory or neurotrophic agents, and even with conventional PD treatment like levodopa, to mitigate side effects like dyskinesia (Table 3) [56].

**Table 3:** Summary of Different GLP-1 Receptor Agonists in Parkinson’s Disease. Table credits: Ruba Ibrahim

GLP-1 RA	Sample Size	Duration	Key Motor Outcomes	Key Non-Motor Outcomes	Disease Modification Potential	Safety/Adverse Events	Relevant Sources
Exenatide	60 patients	48 weeks (plus 12-week washout)	Improved off-medication motor scores (MDS-UPDRS Part III)	Not significantly different	Sustained improvements after washout, suggesting disease modification	Generally well tolerated; GI issues, weight loss (mean 2.6 kg)	^[52]^
Exenatide	194 patients	96 weeks	No significant difference in motor symptom progression	No difference in other measures (e.g., quality of life)	No evidence of slowing progression; no impact on dopamine activity via brain scans	Not specified in snippets, but generally common GI issues for class	^[53]^
Lixisenatide	156 patients	Not specified	May slow the progression of movement-related symptoms	Not confirmed improvements in non-motor symptoms	Stabilized scores, suggesting neuroprotective benefits	More gastrointestinal side effects	^[6]^
Liraglutide	Not specified	Not specified	Did not slow symptom progression	Appeared to improve non-motor symptoms and quality of life	Not specified	Not specified	^[57]^

Timing of intervention: Disease stage matters

The efficacy of GLP-1 receptor agonists (GLP-1RAs) in NDs appears critically dependent on the timing of intervention, specifically the disease stage at which treatment begins.

Utility in Prodromal vs Established Disease

NDs are commonly represented by a lengthy prodromal phase, with little evidence of substantial pathological changes before overt clinical symptoms [26, 31]. Introducing GLP-1 RAs during this early phase is envisioned to have the greatest impact, with a neuroprotective component as well as a disease-modifying component that aims to slow disease progression based on pathological developments as early as when they start to occur (that is, to slow or stop early pathological developments before they result in significant cellular alterations) [13, 26, 27, 31].

There is clinical evidence that supports intervention during this early phase. For example, large-scale real-world data have shown that semaglutide is associated with fundamentally less risk of a first-time diagnosis of AD among patients with type 2 diabetes, suggesting that the drug was beneficial in the earliest stages of neurodegeneration [2, 58, 59]. In particular, ongoing phase 3 trials, such as EVOKE and EVOKE+, are examining oral semaglutide among patients with early stage symptomatic AD, directly targeting its potential disease-modifying properties in this early phase [60].

In contrast, intervention in established disease is limited because neuronal loss in involved areas is significant, with many neurons being lost irreversibly. In this phase, the goals of therapeutics shift somewhat from disease modification to limiting decline or having a symptomatic effect. For example, results from clinical trials of established AD and PDs yield mixed responses. In PD, a phase 2 trial assessing lixisenatide showed decreased progression in motor disability in people with early PD, even if the potential for initial observations to yield confounding explanations exists [46]. However, a larger phase 3 trial of exenatide among patients with moderate severity PD showed no difference in motor symptoms progression [53, 61]. This evidence suggests that GLP-1 RA efficacy strongly depends on the stage of the disease or, more specifically, that the treatment could provide a benefit in an earlier stage [32].

Neuroprotection vs Neurorestoration

An important component of their therapeutic application is delineation between neuroprotection, specifically from neurorestoration, in the context of GLP-1 RA. GLP-1 RAs have value as neuroprotective agents, in protecting existing neurons, preventing cell death, and preventing synaptic integrity through their mechanisms (anti-inflammatory, oxidative stress reduction, improved mitochondrial function, providing trophic effects) [13, 29, 30], GLP-1 RAs are likely to be most effective in prodromal phases, where neurons experience stress, and perhaps are neither damaged or dying, but still fairly intact.

Neurorestoration refers to the repair of structures that have been damaged or the stimulation of new neuron growth. While evidence indicates that GLP-1 RAs can produce neurotrophic effects in preclinical models, widespread neurorestoration has not been achieved in humans with established NDs [13]. Any clinical improvement in late-stage treatment is generally attributed to neuroprotection of surviving neurons. Nonetheless, sustained benefits in some of the early stages of PD, even after treatment has ceased, could highlight its disease-modifying properties. If the intervention occurred early enough, there may have been small restorative effects as well [46, 62].

Optimal Therapeutic Window

Virtually every preclinical and clinical insight seems to speak to the fact that the optimal therapeutic window for GLP-1 RAs will largely occur in the very early stages of NDs [2, 31]. At this point, GLP-1 RAs are better able to be utilized as disease-modifying agents - acting primarily through neuroprotective actions, and most importantly, preventing or significantly delaying irreversible neuronal damage. Through offering neuroprotection to surviving neurons, GLP-1 RAs can preserve cognitive and/or motor function to a greater extent. Moving forward, this is an important consideration for continuing and future clinical trials - the semaglutide EVOKE and EVOKE+ studies, for example, partnered with early-stage or high-risk populations, could help to further confirm this effect [60].

Safety and tolerability in neurodegenerative populations

GLP-1 receptor agonists are generally thought to be safe for treating type 2 diabetes. However, using them to treat neurodegenerative conditions raises new safety issues. The appetite-suppressing properties of GLP-1 RAs related to CNS pharmacodynamics present very valid questions of tolerability, especially in older adults and in those patients where cognitive impairment is present. Randomized controlled trials of GLP-1 RAs (such as exenatide) in a cohort of PD patients have reported a good overall tolerability and indicated that the most common adverse events are mild nausea, appetite suppression, and modest weight loss [53]. For metabolically stable patients, these impacts may not be a concern, but unintentional weight loss may not only inhibit nutritional accommodation but may also exacerbate sarcopenia and clinical decline among the frail older population [63].

Much like in PD, older adults with AD have already been reported to exhibit cachexia and dysregulated appetite, and the appetite-suppressing capabilities of GLP-1 RAs are therefore a particular consideration. Recent highlights from the AD neuroimaging initiative referred to the potential of worsening nutritional deficits in this vulnerable population [64]. Moreover, another study reported the adverse impact of disrupted energy homeostasis on increased neurodegeneration and suggested that even slight reductions in weight could have unfortunate cognitive impacts [65]. Even with these theoretical concerns on the impact of appetite suppression, and a category of cognitive neuropathology, nascent data have indicated that GLP-1 RAs appear to be well tolerated in AD populations, showing very minor CNS-related adverse events in short- and medium-duration trials [66].

Further, we propose that outside of the immediate side effects, we must contemplate the longer-term tolerability of GLP-1 RAs in populations living with ND. There is significant clinical data on the cardiovascular and renal long-term health benefits in metabolic studies, but there is no equivalent cohort of long-duration studies in patients living with NDs. Metabolic studies suggested how important longitudinal safety studies are to potentially show the cumulative effects in the CNS, which are not apparent in shorter terms [67]. As demonstrated by a study done in 2016, it is possible for GLP-1 RA to stabilize brain glucose metabolism for a period of six months, but chronic administration in older brains with the changed responsiveness of receptors, pharmacokinetics under 6-month exposure is still not well researched [68].

Beyond the reality of age alone, elderly individuals are the majority living with NDs, but more complexities of pharmacology are also present in older adults. Changes in gastrointestinal motility, in terms of absorption or removal, changes in hepatic and renal function, changes in responsiveness for CNS receptors, and many other factors all play a role in potential efficacy or side effects. In this context, we expect a proper dosage regimen to require titration based on this number of variables individually. Multiple studies also noted the consideration of potential desensitized receptors and altered neuropeptide signaling that is age-related and that may have implications for the net neuroprotective effect of GLP-1 RAs [2, 69].

In conclusion, while available data suggest that GLP-1 RAs in general are safe and tolerable in patients living with neurodegenerative conditions, there are still very specific considerations related to appetite suppression, frailty, and long-term exposure that could benefit from additional dedicated studies. A call for safety trials of GLP-1 RAs for the elderly, cognitively impaired, and metabolically fragile populations could optimize therapeutic approaches to the inclusion of GLP-1 RAs for maximizing potential neuroprotection and minimizing clinically adverse effects.

Translational potential and barriers to clinical integration

Repurposing GLP-1 RAs for NDs is appealing due to their existing FDA approval for diabetes and obesity, which implies a known safety profile and potentially lower initial development costs. However, this strategy faces significant hurdles. Repurposed drugs still necessitate extensive, high-risk, and costly clinical trials to establish efficacy for new indications, with a single phase 3 AD trial potentially costing hundreds of millions. Trials must also be of long duration given the slow progression of neurodegenerative conditions, and drugs must demonstrate effective blood-brain barrier (BBB) penetration to achieve CNS efficacy. Safety in elderly populations, often with comorbidities and polypharmacy, also requires rigorous assessment [70].

Regulatory processes present a slow and high-attrition pathway [71]. The limited market exclusivity (e.g., 3 years in the US) for a new indication often fails to recoup substantial clinical trial investments, especially if generic versions are available or off-label prescribing occurs [71]. This, coupled with the high cost of new trials and limited remaining patent life, reduces pharmaceutical companies’ incentive to prioritize repurposing projects over novel compounds with greater profitability [70]. Payers also increasingly demand significant clinical benefit or overall cost reduction for reimbursement, further complicating market access [71].

Real-world application introduces additional challenges. Observational studies show lower weight reduction and higher discontinuation rates (20-50% within the first year) for GLP-1 RAs compared to trials, largely due to gastrointestinal side effects and high costs [9]. While weight loss is beneficial for obesity, it can be a concern for frail PD patients. Addressing these adherence, tolerability, and cost-effectiveness issues is crucial for successful clinical integration [61].

Future directions and research gaps

To fully understand the potential of GLP-1 RAs for NDs, future studies must focus on addressing current knowledge gaps and developing a strong evidence base. Overall, there is an urgent need for clinical trials that are larger, longer, and better designed with respect to each GLP-1 RA’s metabolism and dosing, using realistic expectations of therapeutic target exposures and patient populations [66]. For example, it may be useful to consider patient stratification based on metabolic utilization, using non-invasive respiratory exchange ratio, or determining biological disease subtypes to identify patients most likely to experience a positive response [61].

In the short term, there is a need to develop CNS-specific GLP-1 mediators that exhibit improved brain penetration [61]. For instance, we may realize benefits from new multi-agonist formulations as dual agonists (GLP-1/GIP) or even novel triagonists (GLP-1R/GIPR/glucagon receptor) that potentially display greater neuroprotective effects in preclinical studies [72]. Due to the plurality of etiologies for neurodegeneration, organized exploration of rationally designed combination therapies (GLP-1 RAs plus current systemic treatments or other neuroprotective agents) is also a valuable area for future exploration [55]. Additionally, the general neuroprotective mechanisms of GLP-1 RAs could be extended to other ND areas, such as ALS, HD, and frontotemporal dementia, where some ancillary evidence of benefit exists [9].

Limitations

Even with the wealth of information available and the attempts to summarize this available literature, this review has some inherent limitations. First, the vast majority of the literature related to GLP-1 receptor agonists (GLP-1 RAs) in ND is preclinical in nature. Preclinical literature can provide useful mechanistic and proof-of-concept information, but our ability to generalize these findings to human pathophysiology, particularly in complex, chronic, heterogeneous disorders such as AD, PD, and related disorders, is limited.

Second, clinical data are still in their infancy and are frequently limited by small sample sizes, short duration of trials, and heterogeneity of outcome measures. The variation in design and methodology of clinical trials, including, but not limited to, dose, GLP-1 RAs subtype, drug course duration, and cognitive or functional endpoint comparison, complicates any direct comparisons across clinical studies and may mask important subtle effects of efficacy. Many of the trials to date have primarily focused only on early-stage disease processes and prevention models, which leaves the apparent efficacy in moderate-to-advanced stages uncertain.

Third, publication bias is an inherent risk; studies that find a positive effect are more likely to be published than studies with negative or inconclusive results, thereby overestimating efficacy in the literature concerning GLP-1 RAs. Additionally, this review examined only English language publications and studies extracted from selected databases, further leaving the potential for exclusion of relevant data published in languages other than English and other formats.

Fourth, the long-term safety and potential off-target effects of chronic GLP-1 RAs use in neurodegenerative populations, particularly older adults with comorbidities, have yet to be fully delineated in the literature. It is imperative that larger, long-term longitudinal studies continue to evaluate the potential use of GLP-1 RAs for neurodegeneration, with attention to therapeutic potential and durability of response, as well as safety.

Fifth, the co-occurrence of comorbid conditions in neurodegenerative populations may also have an impact on the efficacy and safety of GLP-1 RAs. Many patients with AD, PD, or a related disorder also have type 2 diabetes, cardiovascular disease, or metabolic syndrome, which may influence cognition and motor scores individually. These comorbidities may also mask treatment effects or exaggerate treatment risks as well as influence tolerability, adherence, and side-effect profiles. Future studies of GLP-1 RAs as therapeutic agents for heterogeneous patient populations should consider these variables so that we are able to better understand the efficacy of GLP-1 RAs as options for NDs.

## Conclusions

In conclusion, GLP-1 RAs offer a hopeful multimodal treatment for NDs. This review noted the importance of the treatment’s timing pertaining to early or prodromal states. While there are translational barriers for the clinical applicability of these treatments, including the cost of large clinical trials and safety concerns regarding potential adverse effects in vulnerable patient populations, future, long-term studies across several populations could provide important information about their effectiveness and safety in patient use.
